# Exploring the Hidden Therapeutic Potential of Local Anaesthetics: Antioxidant and Antimicrobial Effects

**DOI:** 10.4274/TJAR.2025.241871

**Published:** 2025-07-24

**Authors:** Berrin Günaydin

**Affiliations:** 1 Gazi University Faculty of Medicine Department of Anaesthesiology and Reanimation, Ankara, Türkiye

**Keywords:** Antimicrobial, antioxidant, articaine, bupivacaine, levobupivacaine, lidocaine, local anaesthetics, pharmacology, ropivacaine

Dear editor,

Nearly two decades ago, Borgeat^1^ reviewed the non-anaesthetic actions of local anaesthetics. While these drugs have been primarily known to block sodium channels for providing anaesthetic/analgesic and antiarrhythmic effects, researchers have also explored several other potential therapeutic uses, including enhanced bowel function after surgery or trauma, protection of the central nervous system, management of chronic neuropathic pain, and possibly reducing cancer recurrence.^1^

There is increasing evidence that local anaesthetics may possess antioxidant properties and interact with reactive oxygen species (ROS). ROS, including peroxyl radicals, hydroxyl radicals, hydrogen peroxide (H_2_O_2_), and superoxide anions (O_2_-), are continuously produced in biological systems as byproducts of mitochondrial metabolism or in response to external stimuli. In healthy cells, ROS are present in low concentrations and play critical roles in defence mechanisms and cellular signalling pathways. These ROS levels are tightly regulated by antioxidant systems, which either directly scavenge the radicals or indirectly modulate their activity. When this balance is disrupted, often due to dysfunction in the antioxidant system, excessive free radical production leads to oxidative stress resulting in cellular damage that includes deoxyribonucleic acid oxidation, lipid peroxidation, protein and enzyme inactivation, and promotion of tumour growth or inflammation.^2, 3, 4^

Early studies demonstrated that some local anaesthetics could positively influence the antioxidant system, mainly through their ability to scavenge free radicals generated either by stimulated human leukocytes or by cell-free systems using luminol chemiluminescence *in vitro*. Based on lidocaine’s known ability to scavenge the O_2_- anion, further study results showed that prilocaine interacted with O_2_-, hypochlorous acid (HOCl), and H_2_O_2_, while articaine reacted with O_2_-, HOCl, and peroxynitrite.^2, 3^ More recently, it was shown that lidocaine exhibits the highest free radical scavenging activity in aqueous environments, but not in lipophilic environments, such as cellular membranes, myelin sheaths, and adipose tissue. This highlights that the scavenging activity of local anaesthetics is influenced by the lipophilicity of the surrounding environment.^4^

Another significant non-anaesthetic effect of local anaesthetics is their antimicrobial activity. Since their introduction as a cornerstone of pain management, numerous studies have explored the antimicrobial properties of local anaesthetics. For example, both bupivacaine and lidocaine have demonstrated bacteriostatic, bactericidal, fungistatic, and fungicidal activities against a wide range of microorganisms.^5^ More recently, three long-acting local anaesthetics-bupivacaine, levobupivacaine, and ropivacaine-demonstrated antifungal activity at both 37 °C and 24 °C. Notably, levobupivacaine (0.75%) and ropivacaine (1%) exhibited antibacterial effects at 37 °C, but not at 24 °C.^5^ These findings suggest that the antimicrobial activity of local anaesthetics may vary with temperature, which warrants further investigation.

Given these emerging therapeutic possibilities, local anaesthetics hold significant promise beyond their conventional use in anaesthesia. The antioxidant activity of local anaesthetics may support their potential use *in vivo*, particularly in conditions linked to free radical damage or antimicrobial effects in regional anaesthesia practice. As research continues to uncover these properties, these drugs are likely to play a larger role in the management of various conditions.
